# Non-necessity of Salt

**Published:** 1854-08

**Authors:** 


NON-NECESSITY OF SALT.—In Europe it is generally supposed that salt is a necessity of life, but here we never found it so. I was once on a riding excursion with Anderson and three other men for six weeks, and a pill box of salt was all we used. The Namaquas occasionally use salt, but they set no store upon it. There is no doubt that people who live on meat and milk would require salt much less than those who live on vegetables; but half the Damaras subsist simply on pig-nuts,—the most worthless and indigestible of food, and requiring to be eaten in excessive quantities to afford enough nourishment to support life. The Hottentots by Walfish Bay, who live almost entirely on the 'Nara gourd, and, who have the sea on one side and salt-springs in front of them, hardly ever take the trouble to collect salt, which they certainly would do if they felt that craving for it which distresses many Europeans. The last fact that I have to mention with reference to salt is, that the game in the Swakop do not frequent the salt-rocks to lick them as they do in America; and I am informed that certain New Zealand tribes not only eat without salt, but actually look upon it with distaste and aversion.—*Galton's Tropical South Africa.*

